# SARS-CoV-2 vaccine-associated-tinnitus: A review

**DOI:** 10.1016/j.amsu.2022.103293

**Published:** 2022-01-25

**Authors:** Syed Hassan Ahmed, Summaiyya Waseem, Taha Gul Shaikh, Nashwa Abdul Qadir, Sarush Ahmed Siddiqui, Irfan Ullah, Abdul Waris, Zohaib Yousaf

**Affiliations:** a Dow University of Health Sciences, Karachi, Pakistan; b Kabir Medical College, Gandhara University, Peshawar, Pakistan; c Department of Internal Medicine, Hamad Medical Corporation, Doha, Qatar

**Keywords:** COVID-19, COVID-19 vaccine, SARS-CoV-2, Vaccine-associated tinnitus, Tinnitus, Ear ringing

## Abstract

The global vaccination drive against severe acute respiratory syndrome coronavirus-2 is being pursued at a historic pace. Unexpected adverse effects have been reported following vaccination, including thrombotic thrombocytopenia, myocarditis, amongst others. More recently, some cases of tinnitus are reported post-vaccination. According to the Vaccine Adverse Events Reporting System (VAERS), 12,247 cases of coronavirus post-vaccination tinnitus have been reported till September 14, 2021. To the best of our knowledge, this is the first review evaluating any otologic manifestation following vaccine administration and aims to evaluate the potential pathophysiology, clinical approach, and treatment. Although the incidence is infrequent, there is a need to understand the precise mechanisms and treatment for vaccine-associated-tinnitus.

## Highlights

• The COVID-19 global vaccination program has significantly reduced hospitalization and deaths due to infection.
• According to VAERS, 12,247 cases of coronavirus post-vaccination tinnitus have been reported till September 14, 2021.
• This review highlights the potential mechanism, diagnosis and management of COVID-19 vaccine associated tinnitus.
• Corticosteroids were the first line treatment options for SARS-CoV-2 vaccine-associated tinnitus.

## 1 Introduction

The SARS-CoV-2 virus has infected approximately 225 million people globally, resulting in 4.6 million deaths [1]. It commonly manifests as fever, dry cough, shortness of breath, fatigue, and myalgias. However, it can also lead to severe complications like pneumonia, leukopenia, kidney failure, myocardial involvement, and central nervous system (CNS) disorders [2].

Vaccinations are arguably the most effective preventive tool against SARS-CoV-2. In August 2020, Russia became the first country to register Sputnik V, a coronavirus vaccine based on human adenovirus vectors rAd26 and rAd5 developed by the Gamaleya national center of epidemiology and microbiology. However, this vaccine was approved without phase III trials, raising concerns over its safety [3].

The currently available vaccines underwent clinical trials and were approved after demonstrating an acceptable safety profile and efficacy [4]. To date, 5.5 billion vaccine doses have been administered [1]. The adverse effects of vaccines are mostly mild and transient, commonly including pain at the injection site, pyrexia, headache, myalgias, fatigue, chills [5] and dermatologic manifestations like Pityriasis Rosea [6]. However, severe complications like anaphylaxis [7], vaccine-induced immune thrombotic thrombocytopenia [8], myocarditis [9] have also been reported. The adverse effects of vaccine are markedly outweighed by their beneficial effects, in decreasing hospital admissions and deaths due to the SARS-CoV-2 [10,11].

Investigations of the otologic manifestations of the SARS-CoV-2 suggest the incidence of tinnitus, hearing loss, sensorineural hearing loss (SNHL), otalgia, amongst others. However, only association with tinnitus and hearing loss were statistically significant [12]. More recently, cases of tinnitus presented following both vector-based and mRNA SARS-CoV-2 vaccines [13,14]. According to the Vaccine Adverse Event Reporting System (VAERS), 12,247 cases of tinnitus post-coronavirus vaccination have been reported [15].

Tinnitus is an otologic symptom characterized by a conscious perception of sound without an external auditory stimulus. The prevalence varies from one population subset to another [16]. The study by Jong Kim et al., which employed data from the Korean National Health and Nutrition Examination survey, reported tinnitus prevalence to be 20.7% among adults, i.e., 20- to 98-year-old [17]. The National Health and Nutritional Examination survey data indicated a prevalence of 16.5% among the overall population and 6.6% among Asian Americans [18]. Along with varying prevalence, it has also been associated with a wide range of risk factors including male gender, hearing impairment, ear infections, stress, unemployment, military services, dyslipidemia, osteoarthritis, rheumatoid arthritis, asthma, depression, thyroid disease, noise exposure, history of head injury and numerous others [17,19].

Herein, we review the association between SARS-CoV-2 vaccines and tinnitus. This review aims to evaluate the potential pathophysiology, clinical approach to diagnosis and management of post-vaccination tinnitus.

## 2 Literature search, data extraction, and results

**Fig. 1:**
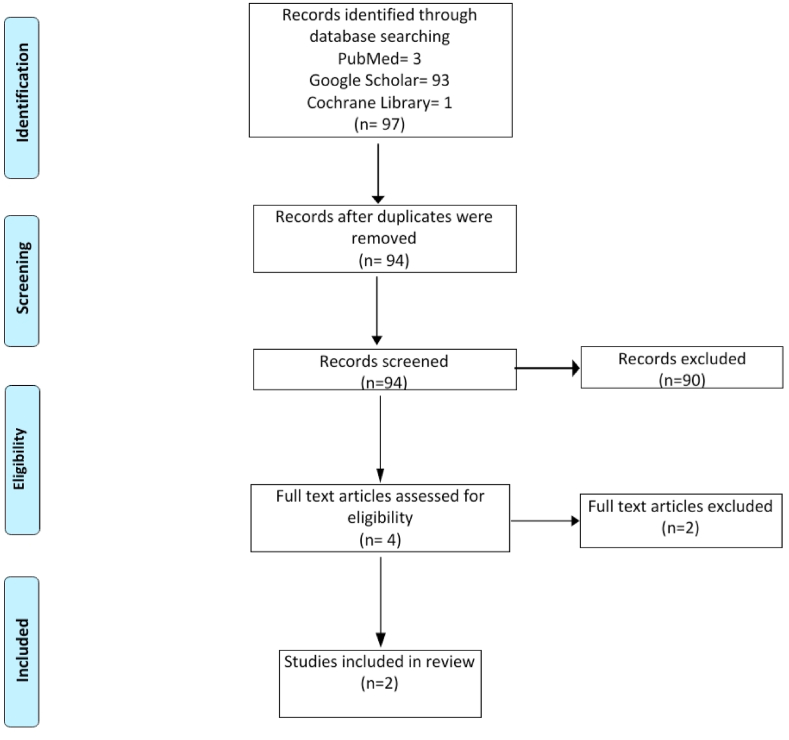
Preferred Reporting Items for Systematic reviews and Meta-Analyses (PRISMA) flowchart.

Two independent authors (SHA, TGS) conducted a thorough literature search over PubMed, Cochrane Library, and Google Scholar from inception till September 12, 2021, without any language restriction. To achieve comprehensive results, search string comprised of keywords, “SARS-CoV-2 Vaccine”, “Coronavirus Vaccine,” “Corona Vaccine,” “COVID-19 Vaccine”, “Tinnitus,” “Ear Ringing,” “Otologic Manifestations,” and separated by BOOLEAN operators “OR” and “AND.” All relevant case reports, case series, cohort studies, editorials, and correspondences were reviewed. Grey literature and bibliographies of the relevant articles were also screened. Results of the literature search are summarized in Fig. 1. The work has been reported in line with the Preferred Reporting Items for Systematic Reviews and Meta-Analyses (PRISMA) 2020 criteria [20].

**Fig. 2:**
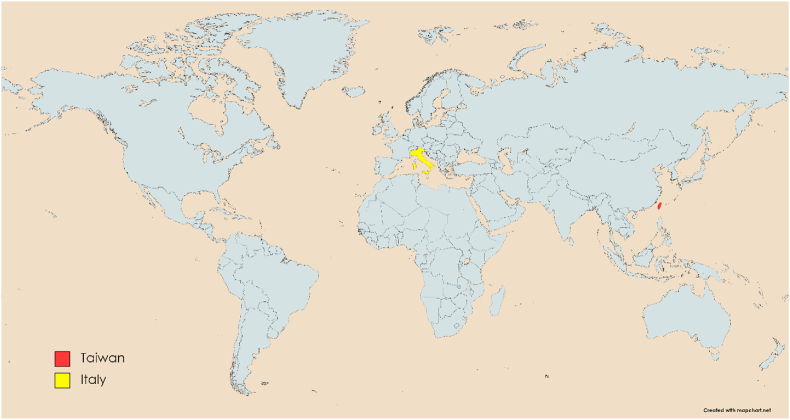
Geographical distribution of reported cases.

Ultimately, two studies [13,14] (case report and case series) were retrieved for inclusion in the review. The studies comprised data from four patients (three males and one female) with a mean age of 41.8 ± 12.6 years. The following figure (Fig. 2) demonstrates the geographical locations where these cases were reported. Out of the four reported cases, three presented in Italy, while one was reported from Taiwan. Along with these findings, future research may enable us to predict the gender, age groups, and geographical locations that may leave certain individuals more susceptible to COVID-19 vaccine-associated tinnitus than others.

Following studies selection, two independent authors (SW, NAQ) retrieved all the relevant data comprising of author's name, patient's age, and sex, past medical history, vaccine administered, time from dose administration till the onset of symptoms, presenting complaint, laboratory findings, treatment interventions, and outcome into a table. All significant findings are summarized in Table 1. Any discrepancies were resolved by discussion with a third reviewer (SS).

**Table 1:** A tabulation of the outcomes of literature review.

Author	Age Sex	Past Medical History	Vaccine Administered	Time from Vaccination to Onset of symptoms	Presenting Complaint	Investigations	Treatment	Outcome
Tao-Tseng et al. [14]	37 y/o Male	Glaucoma is treated with latanoprost and brimonidine eye drops	ChAdOx1 nCoV-19 AstraZeneca (1st dose)	5 h	Intermittent, high pitch, right ear tinnitus, high fever with chills and myalgias. It progressed to continuous high pitch and intermittent low pitch tinnitus.	THI = 28 (5 h post vaccination) THI = 46 (after visiting emergency) Audiometry test on 1st May revealed normal PTA and short SiSi THI (post-treatment) = 0	Single-dose of 10 mg IV dexamethasone and 3 × 5 mg oral prednisone daily for 3 days.	Recovered on day 4
Parrino et al. [13]	37 y/o Female	Glaucoma, undifferentiated connective tissue disease, and transient tinnitus due to acute otitis media 20 years previously	BNT162b2 mRNA-vaccine Pfizer (1st dose)	7 h	Right ear tinnitus, short-term dizziness, pain at the injection site.	Otoscopy investigation was normal. PTA revealed normal bilateral hearing with slight asymmetry on the right ear THI = 90/100 Psychoacoustic Measures of Tinnitus = 20 dB pure tone at 10,000 Hz THI (post-treatment) = 78/100	30 mg Deflazacort daily given orally for first 5 days followed by 15mg/daily dose for next 5 days.	Recovering
Parrino et al. [13]	63 y/o Male	Bilateral symmetrical mild high frequencies SNHL, chronic gastritis, extrinsic asthma, and reactive depression for which he had undergone psychotherapy	BNT162b2 mRNA-vaccine Pfizer (1st dose)	20 h	Left tinnitus associated with hyperacusis and dysacusis and local pain at the injection site	Otoscopy examination was normal. PTA revealed slight threshold worsening on the left ear Psychoacoustic Measures of Tinnitus = white noise of 25 dB intensity THI = 76/100 THI (after 7 days) = 36/100	Corticosteroid therapy was proposed, but the patient refused.	Recovering
Parrino et al. [13]	30 y/o Male	Hashimoto thyroiditis	BNT162b2 mRNA-vaccine Pfizer (2nd dose)	6 days	Left tinnitus, hyperacusis, dysacusis. Reported fever, nausea, and local pain after dose administration that was treated with 1 × 1000 mg acetaminophen	Otoscopy was normal PTA showed normal bilateral hearing. THI = 78/100 THI (post-treatment) = 6/100	10 days course of oral prednisone at 50 mg/day for first 4 days followed by 25 mg/day for the next 3 days and 12.5 mg/day for the last 3 days.	Recovered

THI: Tinnitus Handicap Inventory, PTA: Pure Tone Average, SiSi: Short increment Sensitivity index, SNLH: sensorineural hearing loss.

## 3 COVID-19 vaccines and their characteristics

Most of the current COVID-19 vaccines use the genetic code of spike protein to stimulate a protective immune reaction against coronavirus. The viral vector vaccines (AstraZeneca, sputnik, Janssen) incorporate spike protein gene into adenovirus DNA, which induces spike protein formation and hence antibodies, conferring protection against the virus. Conversely, mRNA vaccines (Pfizer, Moderna) deliver messenger RNA for spike protein into the host cells, stimulating a protective response [21]. Another category of COVID-19 vaccines (Sinopharm, Sinovac) employs a weakened or attenuated virus, capable of replication but not potent enough to cause the disease itself [22].

Moreover, research done after SARS-CoV-1 indicated the protective and long-lasting effect of T-cell immunity. The transfer of T-cells led to a swift viral clearance and disease elimination [23,24] Unlike antibody response, T cell memory can last longer as seen in SARS-CoV-1 when the immunity was even detected 4 years after the infection. Especially, Regulatory T cells play a vital role in resolving the infection, confirmed from the fact that they were found to be risen in COVID-19 patients [25]. Along with them, circulating follicular T helper cells have been seen in individuals with COVID-19. They play a major role in representing antibody response to infection. Hence, despite no vaccine currently offering the T-cell response to COVID-19, there is a room to further investigations.

Listed in Table 2 are some of the most common vaccines currently used to counter the pandemic and their characteristics including mechanism of action, dosage, time between dosages, efficacy, general and serious adverse effects. What is of immense concern is the fact that despite a vast previous knowledge on T-cell immunity, none of the marketed vaccine is using it as a mechanism of their action. Hence, leaving room for further investigations.

**Table 2:** Table 2: Characteristics of COVID-19 vaccines.

Vaccine	Manufacturer & Country	Mechanism of Action	Doses – Time Between Doses	Efficacy	Adverse Effects	Serious Adverse Effects
BNT162b2	BioNTech, Fosun Pharma, Pfizer – America and Germany	RNA vaccine [26,27]	Two doses - 3 weeks [26]	100% against severe disease as per CDC, 93% against severe disease as per FDA [26]	Redness, Swelling, Headache, Muscle pain, Chills, Fever, Nausea, Tiredness [28]	Lymphadenopathy, paroxysmal ventricular arrhythmia, syncope, and right leg paresthesia [29], heart inflammation in young adults [26]
mRNA-1273	Moderna- U.S. and Switzerland	RNA vaccine [26]	Two doses - 4 weeks [26]	>90% [26]	Pain, swelling, redness, fever, fatigue, headache, vomiting, arthralgia, myalgia, urticaria [28]	Bell's Palsy, facial swelling [27]
ChAdOx1 nCoV-19/AZD1222	AstraZeneca (University of Oxford) – U.K.	Viral vector vaccine [27]	Two doses −4 to 12 weeks [26]	76% (phase III trials) [26]	Redness, myalgias, arthralgias, and headache [27]	Pulmonary embolism, Thromboembolism [27]
Ad26.COV2.S	Johnson & Johnson -U.S.	Viral vector vaccine [26]	A single dose [26]	72% [26]	Pain, redness, and swelling at the injection site [27]	Rare and Severe blood clots [27]
Ad5-nCoV	Cansino - China	Viral vector vaccine [30]	A single dose [30]	65.7% [30]	Fever, redness, and pain [30]	Not reported [30]
Coronavac	Sinovac – China	Inactivated Virus [31]	Two doses – 2 to 4 weeks [32]	51% [32]	Pain on injection [31]	Acute hypersensitivity with the manifestation of urticaria [31]
BBIBP-CorV	Sinopharm - China	Inactivated Virus [33]	Two doses – 2 to 3 weeks apart, followed by a booster dose in Age group >18 years [33].	79% [33]	Pain at the vaccination site, fatigue, lethargy, headache, and tenderness [34]	
Gam-COVID-Vac/Sputnik V	Gamaleya Research Institute of Epidemiology and Microbiology - Russia	Viral Vector Vaccine [35]	2 doses, 3 weeks apart [35]	91.6% [35]	Mild pain at the injection site, fever, headache, fatigue, and muscle aches [36]	

WHO: World Health Organization; CDC: Center for Disease Control and Prevention; FDA: U.S. Food and Drug Administration.

Moreover, all the listed vaccines include the ones currently, accepted in many countries throughout the world. With frequent introduction of numerous vaccines in the market to combat the pandemic, there is a definite need to evaluate their characteristics in comparison and the better ones shall be publicly made available.

## 4 Pathophysiology

Tinnitus is defined as intermittent or continuous, unilateral or bilateral, pulsatile or non-pulsatile, acute or chronic, and subjective or objective [37,38]. There are several classifications categorizing tinnitus into numerous types, with each type associated with multiple potential etiologies. It can result from a lesion in the auditory pathway. Potential etiologies may include otitis externa, cerumen impaction, otosclerosis, otitis media, cholesteatoma, vestibular schwannoma, Meniere's disease, colitis, neuritis, and ototoxic drugs [37,38]. The character of tinnitus can vary based on etiology. Furthermore, certain non-otologic conditions like vascular anomalies, myoclonus, and nasopharyngeal carcinoma can also contribute. Despite several cases of tinnitus being reported post-SARS-CoV-2 vaccination, the precise pathophysiology is still not clear.

### 4.1 Molecular mimicry

Based on the mechanisms behind other COVID-19 vaccine-induced disorders (38, 39) and the phenomenon of molecular mimicry [41], a cross-reactivity between anti-spike SARS-CoV-2 antibodies and otologic antigens is a possibility. The heptapeptide resemblance between coronavirus spike glycoprotein and numerous human proteins further supports molecular mimicry as a potential mechanism behind such vaccine-induced disorders [41]. Several autoimmune conditions, including vaccine-induced thrombotic thrombocytopenia (VITT) [8] and Guillain-Barré syndrome (GBS) [40], have been reported following coronavirus vaccination. Anti-spike antibodies may potentially react with antigens anywhere along the auditory pathway and initiate an inflammatory reaction involving the tympanic membrane, ossicular chain, cochlea, cochlear vessels, organ of Corti, etc. Therefore, understanding the phenomenon of cross-reactivity and molecular mimicry may be helpful in postulating potential treatment behind not only tinnitus but also the rare events of vaccination associated hearing loss and other otologic manifestations [42]. Moreover, serologic investigations may play a role in understanding the underlying mechanism. Specific findings, such as raised anti-platelet factor 4, have been reported in cases of VITT post-COVID-19 vaccination [39].

### 4.2 Autoimmune reactions

Antibodies can form complexes with one or more antigens leading to a type III hypersensitivity reaction. Deposition of circulating immune complexes and vestibule-cochlear antibodies can play a role in autoimmune inner ear disease [43,44]. Incidence of pre-existing autoimmune conditions like Hashimoto thyroiditis and gastritis in patients, as shown in Table 1, further leaves patients prone to immune dysfunction and thus abnormal immune responses [13]. However, future research should investigate the incidence of post-vaccination tinnitus in individuals with autoimmune diseases with a suitable control as all the currently reported patients were known cases of such conditions. Moreover, several potential genes, including Glial cell Derived Neurotrophic Factor (GDNF), Brain Derived Neurotrophic Factor (BDNF), potassium recycling pathway genes, 5-Hydroxytryptamine Receptor 7 (HTR7), Potassium Voltage Gated Channel Subfamily E Regulatory Subunit 3 (KCNE3), and a few others, have been studied to understand the underlying mechanism. However, the evidence is still insufficient to draw any conclusion [45]. Therefore, genetic predisposition and immunologic pathways may play a role in post-vaccination-tinnitus.

### 4.3 Past medical history

Literature suggests a relationship between glaucoma and tinnitus, with glaucoma patients having 19% increased odds for tinnitus than in patients without it [46]. The mechanism linking these disorders is ambiguous, but vascular dysregulation may play a significant role in causing both disorders. Nitric oxide (NO) production inhibition is a potential mechanism [46]. NO is a regulator of intraocular pressure (IOP), thus linking defects in the nitric oxide guanylate cyclase (NO-GC) pathway with glaucoma [47]. Furthermore, diminished jugular vein NO levels have been reported in tinnitus patients, leading to the reduced blood supply to the ears [46]. As shown in Table 1, two of the reported cases had pre-existing glaucoma. Therefore, any potential association between vaccines and NO dysregulation should be investigated. Certain COVID-19 vaccines have been associated with vaccine-induced thrombotic thrombocytopenia [8]. Developing thrombus can reduce the blood supply to the ear and increase the probability of developing tinnitus. The existing literature lacks articles investigating associations between vaccines and NO levels. Therefore, the association of vaccines with NO deficiency in genetically susceptible patients should be investigated. Lastly, the association between vaccines and other vascular dysregulations must also be evaluated, as such abnormalities can disrupt laminar blood flow and cause pulsatile tinnitus [48].

### 4.4 Ototoxicity

Numerous drugs and chemical substances have been reported as ototoxic, causing damage to the auditory pathway and cochlear hair cells. Exposure to such agents, including aminoglycosides, vancomycin, platinum-based anticancer drugs, loop diuretics, quinine, toluene, styrene, lead, trichloroethylene, and others, may lead to tinnitus, hearing loss, and other otologic manifestations [37,49]. The mechanisms behind ototoxicity are not fully understood but may involve chemical and electrophysiological alterations in the inner ear structures and the eighth cranial nerve. Certain agents, including loop diuretics, incite such symptoms by inhibiting endolymph production from stria vascularis, whereas drugs like aminoglycosides and cisplatin are directly toxic to the hair cells the organ of Corti. Meanwhile, Non-Steroidal Anti-Inflammatory Drugs (NSAID) induce ototoxicity by reducing cochlear blood flow and alterations in the sensory cell functions [50]. Hence, the possibility of one or more vaccine components exerting ototoxic effects cannot be written off and requires attention.

Furthermore, the current literature also proposes certain risk factors associated with drug-induced ototoxicity. For example, age, hypoalbuminemia, and uremia significantly increase the risk of developing NSAIDs induced ototoxicity. Similarly, erythromycin-related ototoxicity is more commonly associated with hepatic and renal failure, increasing age and female gender [50]. Therefore, genetic predispositions and associated conditions may also play a significant role in determining the development of vaccine-induced tinnitus. As shown in Table 1, most of the cases reported till now were transient, which may be accountable to past administration of offending agents as seen in cases of erythromycin, aminoglycosides, vancomycin, and NSAIDs associated ototoxicity, which resolved upon early discontinuation of the inciting agent [50].

### 4.5 Psychological conditions

Anxiety-related adverse events (AEFI) following vaccination, defined by WHO, “a range of symptoms and signs that may arise around immunization that are related to anxiety and not to the vaccine product, a defect in the quality of the vaccine or an error of the immunization program” [51], have been witnessed in around 25% COVID-19 vaccination cases in India, as reported by Government of India, Ministry of health and family welfare, immunization division [52]. These responses may include vasovagal mediated reactions, hyperventilation mediated reactions, and stress-related psychiatric reactions or disorders [53]. Loharikar et al. [54], in their systematic review, reported common symptoms of it to be dizziness, headache, and fainting with rapid onset after vaccination. There are several speculations on the causative agents behind AEFIs after immunization. Since most of the vaccines are delivered through needles, it may be possible that trypanophobia, affecting at least 10% of the population around the globe [55], may trigger stress, hence leading to a stress-mediated response. Moreover, hearing or witnessing someone else's sickness can lead to reporting similar symptoms, known as psychogenic illness, as reported by Blaine Ditto et al. [56]. Hence, a possible connection can exist between people's presumption and social media misinformation, leading to anxiety and possible adverse reaction.

Vaccine hesitancy, defined as a “delay in acceptance or refusal of vaccination despite the availability of vaccination services” [57], is a complex behavior, and the most common cause of it usually includes perceived risks vs. benefits, religious beliefs, and lack of knowledge [58]. People with vaccine hesitancy may have pre-assumed beliefs. Hence, after getting vaccinated, there is a chance of facing AEFIs, with symptoms constellating stress. Numerous studies have demonstrated anxiety and stress as risk factors for tinnitus [17,19]. In one of the reported cases [13], the patient had a history of reactive depression. Therefore, the incidence of anxiety and stress disorders also need to be explored, with a particular emphasis on vaccine-related anxiety, as a potential cause of tinnitus developing post-vaccination.

### 4.6 Overview

While several suggested hypotheses exist, the precise mechanism behind vaccine-induced tinnitus remains undetermined, leaving room for future studies. Furthermore, as shown in Table 1, two reported cases had a medical history of otologic conditions involving recovered tinnitus and SNHL. Therefore, the possibility of vaccines aggravating underlying otologic disorders and exacerbating any morphologic damage also needs to be explored. Lastly, the character of tinnitus, including subjective or objective, intermittent or continuous, and pulsatile or non-pulsatile, can also give beneficial insight into understanding the involved sights and underlying mechanisms.

## 5 Clinical approach and management

To start the treatment regimen, it is crucial to determine a well-established diagnosis for Tinnitus. For this purpose, a well-focused and detailed history and examination are necessary [38]. In case of vaccine-induced tinnitus, vaccine administered, days since dose administered to the onset of symptoms, and any other adverse effects experienced must be further added. Additionally, a particular emphasis must be placed on pre-existing health conditions, specifically autoimmune diseases like Hashimoto thyroiditis, otologic conditions like SNHL, glaucoma, and psychological well-being. All the reported patients presented with a history of one or more of the aforementioned disorders, as shown in Table 1. However, any such association has not yet been established and requires further investigation to be concluded as potential risk factors for vaccine-induced tinnitus. Routine cranial nerve examination, otoscopy, Weber's test, and Rinne test, that are used for tinnitus diagnosis in general [38], may also be used for confirmation of the disorder post-vaccination. Due to the significant association between tinnitus and hearing impairment [59], audiology should be performed as well.

Tinnitus handicap inventory (THI), a reliable and valid questionnaire to evaluate tinnitus-related disability [60], is recommended by the tinnitus research initiative (TRI) [61]. To date, it has been translated into numerous languages and is being used across the globe. In THI, the scores of 0, 2, and 4 are assigned to no, sometimes, and yes, respectively, to answer a subset of questions. The scores can vary from 0 to 100, with higher scores indicating a more significant disability. Based on scores, the patients can be classified into five categories: Scores ranging between (1) 0 to 16 indicate no handicap, (2) 18 to 36 indicate mild handicap, (3) 38 to 56 indicate moderate, (4) 58 to 76 indicate severe handicap and (5) 78 to 100 indicate catastrophic handicap [62]. This scale can be employed to evaluate both the severity of the condition and therapeutic response, as reported in the included studies [13,14].

While the treatment options for non-vaccine-induced tinnitus show a significant degree of variance, corticosteroids were the lead treatment choice for SARS-CoV-2 vaccine-induced tinnitus, as reported in both the included studies [13,14]. Based on the results, Tseng et al. [14] recommend immediate use of steroids for sudden onset tinnitus post-coronavirus vaccination. The reason may lie in their underlying immunosuppressive mechanism. After entering the cell, Corticosteroid forms a steroid-receptor complex in the cytoplasm, which then modifies transcription by incorporating itself into DNA. Hence playing their role in synthesizing or inhibiting certain proteins. A well-known protein synthesized by them is lipocortin, which inhibits Phospholipase A2, ultimately inhibiting arachidonic acid (AA) which leads to hampered Leukotrienes and Prostaglandins production. It also impedes mRNA that plays role in interleukin-1 formation [63] as well as sequestrate CD4^+^ T-lymphocytes in the reticuloendothelial system, all building up and leading to immunosuppression [64].

Although two out of four patients showed improvement following drug administration, the efficacy of steroid therapy is yet to be investigated in larger populations.

There is also a dire need to perform trials for other pharmacological interventions that can be administered in post-vaccine tinnitus. Numerous non-pharmacological (counseling, tinnitus retraining therapy, sound therapy, auditory perceptual training) as well pharmacological interventions (sodium channel blockers, anti-depressants, anti-convulsant, benzodiazepines, and several others) for treatment of tinnitus have been evaluated [16,65], however, there is insufficient data for tinnitus following vaccination, despite that vaccine-induced tinnitus have also been reported after hepatitis B, rabies, measles and (influenza A virus subtype) H1N1 vaccines, associated to Sensorineural hearing loss (SNHL) [66].

Thereby, deeming high-quality trials evaluating the efficacy of conventional treatment necessary. Lastly, the transient nature also requires special attention, as one of the patients recovered without any medication [13].

## 6 Adverse effects monitoring

Although the COVID-19 vaccines were approved after rigorous testing and trials, the center for disease control and prevention (CDC) has taken numerous initiatives to ensure a highly intensive safety monitoring program to determine potential adverse effects that may not be reported during clinal trials. Several vaccine safety monitoring systems are being employed, including the VAERS, v-safe, clinical immunization safety assessment (CISA) program, vaccine safety datalink (VSD), and a few others. This wide range of systems allows patients, attendants, and healthcare workers to report any side effects they have been experiencing following SARS-CoV-2 vaccination. CDC and vaccine safety experts evaluate all the reports regularly and assess vaccines safety on their basis [67]. Investigations into reported side effects are conducted to ensure vaccines safety, as was observed following cases of thrombotic thrombocytopenia, which led to a temporary ban on two vaccines and were only lifted once the vaccines demonstrated an acceptable safety profile. With already established benefits and such critical safety monitoring, the COVID-19 global vaccination program must be supported and appreciated for prioritizing public safety. However, such reporting systems may be more useful if there was a way to determine if the reported adverse events were vaccine-induced, exacerbated following vaccination, or due to some underlying pathology.

## 7 Conclusion

This review scrutinizes the currently available literature and highlights potential pathophysiology and clinical approaches to diagnose and manage vaccine-induced tinnitus. Although the incidence of COVID-19 vaccine-associated tinnitus is rare, there is an overwhelming need to discern the precise pathophysiology and clinical management as a better understanding of adverse events may help in encountering vaccine hesitancy and hence fostering the COVID-19 global vaccination program. Despite the incidence of adverse events, the benefits of the SARS-CoV-2 vaccine in reducing hospitalization and deaths continue to outweigh the rare ramifications.

## 8 Limitations

This study carries some limitations. Firstly, given the limited number of cases reported, there is an imperative need to overcome the paucity of data and evaluate the impact of different COVID-19 vaccines, type of tinnitus, response to conventional treatment options, and reversible nature of the condition. Secondly, all the patients evaluated reported substantial past medical history and carried a high risk of immune dysregulation; therefore, the role of genetic predisposition and underlying conditions requires special surveillance, which can help redefine vaccine administration criteria to avoid any further cases.

## Ethical approval

NA.

## Sources of funding

The authors have no funding source to declare.

## Author contributions

SHA, IU: Study concept or design.

SHA, SW, TGS, NAQ, SAS, IU and AW: Data collection, data analysis or interpretation, writing the paper.

ZY: Critical revision of the article.

## Registration of research studies

1. Name of the registry: NA.

2. Unique Identifying number or registration ID: NA.

3. Hyperlink to your specific registration (must be publicly accessible and will be checked): NA.

## Guarantor

Irfan Ullah.

Kabir Medical College, Gandhara University.

Peshawar, Pakistan Irfanullahecp2@gmail.com

Zohaib Yousaf

Department of Internal Medicine, Hamad Medical Corporation, Doha, Qatarzohaib.yousaf@gmail.com

## Consent

NA.

## Conflict of interests

The authors declare that there is no conflict of interests.

## Provenance and peer review

Not commissioned, externally peer-reviewed.

## Declaration of competing interest

None.
